# Outcome of total knee replacement following explantation and cemented spacer therapy

**DOI:** 10.3205/iprs000091

**Published:** 2016-03-24

**Authors:** Mohamed Ghanem, Dirk Zajonz, Juliane Bollmann, Vanessa Geissler, Torsten Prietzel, Michael Moche, Andreas Roth, Christoph-E. Heyde, Christoph Josten

**Affiliations:** 1 Department of Orthopaedic Surgery, Traumatology and Plastic Surgery, University Hospital Leipzig, Leipzig, Germany; 2 Department of Diagnostic and Interventional Radiology, University Hospital Leipzig, Leipzig, Germany

**Keywords:** periprosthetic infection, endoprosthesis infection, cemented spacer therapy, total knee replacement

## Abstract

**Background:** Infection after total knee replacement (TKR) is one of the serious complications which must be pursued with a very effective therapeutic concept. In most cases this means revision arthroplasty, in which one-setting and two-setting procedures are distinguished. Healing of infection is the conditio sine qua non for re-implantation.

This retrospective work presents an assessment of the success rate after a two-setting revision arthroplasty of the knee following periprosthetic infection. It further considers drawing conclusions concerning the optimal timing of re-implantation.

**Patients and methods:** A total of 34 patients have been enclosed in this study from September 2005 to December 2013. 35 re-implantations were carried out following explantation of total knee and implantation of cemented spacer. The patient’s group comprised of 53% (18) males and 47% (16) females. The average age at re-implantation time was 72.2 years (ranging from 54 to 85 years). We particularly evaluated the microbial spectrum, the interval between explantation and re-implantation, the number of surgeries that were necessary prior to re-implantation as well as the postoperative course.

**Results:** We reported 31.4% (11) reinfections following re-implantation surgeries. The number of the reinfections declined with increasing time interval between explantation and re-implantation. Patients who developed reinfections were operated on (re-implantation) after an average of 4.47 months. Those patients with uncomplicated course were operated on (re-implantation) after an average of 6.79 months. Nevertheless, we noticed no essential differences in outcome with regard to the number of surgeries carried out prior to re-implantation. Mobile spacers proved better outcome than temporary arthrodesis with intramedullary fixation.

**Conclusion:** No uniform strategy of treatment exists after peri-prosthetic infections. In particular, no optimal timing can be stated concerning re-implantation. Our data point out to the fact that a longer time interval between explantation and re-implantation reduces the rate of reinfection. From our point of view, the optimal timing for re-implantation depends on various specific factors and therefore it should be defined individually.

## Zusammenfassung

Die periprothetische Infektion nach Kniegelenksendoprothesen (K-TEP) ist eine der schwerwiegenden Komplikationen, die mit einem möglichst effektiven Konzept therapeutisch verfolgt werden muss. In den meisten Fällen bedeutet dies den Wechsel der Endoprothese, wobei einzeitige und zweizeitige Verfahren unterschieden werden. Zwingende Voraussetzung für die Re-Implantation ist die Infektsanierung.

Das Ziel der vorgestellten retrospektiven Arbeit ist die Vorstellung der Erfolgsrate von Re-Implantationen nach periprothetischen K-TEP-Infektionen bei zweizeitigen Eingriffen. Sind dabei Rückschlüsse hinsichtlich des optimalen Zeitpunktes der Re-Implantation möglich?

**Patienten und Methoden:** Es konnten insgesamt 34 Patienten von September 2005 bis Dezember 2013 eingeschlossen werden, bei denen 35 Re-Implantations-Operationen nach Knie-Endoprotheseninfektionen mit erfolgtem Ausbau und Spacerbehandlung durchgeführt wurden. Das Patientenkollektiv umfasste 53% (18) männliche und 47% (16) weibliche Patienten. Das Durchschnittsalter zum Re-Implantations-Zeitpunkt lag bei 72,2 Jahren (54 bis 85 Jahre). Ausgewertet wurden unter anderem das Erregerspektrum, das Intervall zwischen Ausbau und Re-Implantation, die Anzahl an nötigen Revisionen bis zur Re-Implantation und die weiteren postoperativen Verläufe.

**Ergebnisse:** Bezogen auf die 35 periprothetischen Infektionen kam es nach der Re-Implantation bei 31,4% (11) zu Re-Infektionen. Mit zunehmender Zeitspanne zur Re-Implantation sank die Anzahl der Re-Infektionen. (Patienten mit Re-Infektionen wurden durchschnittlich nach 4,47 Monaten reimplantiert, diejenigen Patienten mit bis dato komplikationslosen Verläufen nach 6,79 Monaten.) Die Anzahl der erfolgten Revisionen bis zur Re-Implantation hatte in unserer Arbeit keinen Einfluss auf den Outcome. Bewegliche Platzhalter hatten tendenziell bessere Erfolgsraten als intrameduläre Spacer.

**Schlussfolgerung:** Es existiert keine einheitliche Behandlungsstrategie nach peri-prothetischen Infektionen. Insbesondere kann kein optimaler Re-Implationszeitpunkt genannt werden. Unsere Daten weisen darauf hin, dass sich eine größere Zeitspanne zur Re-Implantation als vorteilhaft für die Re-Infektionsrate erweist. Aus unserer Sicht ist der optimale Re-Implantationszeitpunkt von diversen spezifischen Faktoren abhängig und somit individuell festzulegen.

## Introduction

Arthroplasty and revisionarthroplasty of the knee are frequent interventions in the industrialized nations with a clearly rising trend. In 2013, 127,192 primary total knee replacement (TKR) and 17,428 TK-revision surgeries were performed in Germany ([1], p. 167-72). Due to the increasing numbers of operations, complications continued to rise. Following aseptic loosening and instability, infection is the third leading cause for revision surgery. Assuming the rate of infection that is reported in literature of 0.4–4%, considered at best with 1.5% of primary TKRs, so you can almost consider 2,000 infections after TKR per year in Germany [5], [13], [15], [27], [28], [33], [34]. Here, the rate of infection after revision surgery (ca. 5%) and the rate of infection after re-implantation (ca. 15–20%) were not taken into account, which is why one must speak of a significantly greater number [11], [26]. The periprosthetic infection is the most serious complication after arthroplasty that can even have a lethal outcome in some cases, and thus represents a significant risk to the patient.

The most commonly identified microorganisms are gram-positive cutaneous bacteria such as staphylococci (*Staph. aureus* and *Staph. epidermidis*) [5], [21], [31]. The increasing incidence of multiresistant pathogens (MRE) is described as a significant problem in this case [5], [21], [32].

In case of a periprosthetic infection, a consistent therapeutic concept must be adopted in order to re-establish a pathogen-free situation. The preservation of the artificial joint can only be considered in acute infection with particular staphylococci and streptococci or subcutaneous abscesses [11], [34]. Early action, the radical nature of debridement and knowledge of the germ nature are crucial to the success of this method [11], [34]. Revision surgery with replacement of the artificial joint is carried out much more frequently. Distinction can be made between a one-stage and two- or multi-stage procedures.

Some authors describe advantages of the one-stage procedure such as the reduction of physical and mental stresses of a second major intervention and the avoidance of uncertainty and disability between the operations [11], [10]. The costs are up to 24% lower in a one-stage procedure than in a two-stage approach, giving financial and economic dimensions to the chosen strategy [23]. It is questionable, however, whether complete remission of infection can be achieved.

The most significant advantage of two- or multi-stage approach can thus be seen in the higher rate of eradication of infection compared to single-stage approach [30]. In addition, an unknown germ situation and possible resistance to antibiotics favor the two-stage approach, as this provides the opportunity to have histological and microbiological samples [10], [34]. Further, the extent of the systemic effects of infection or the occurrence of systemic infection or sepsis seems to be less likely [11], [22], [26]. Another advantage is to facilitate the planned revision procedure in case of persistence of infection because neither an implant nor cement is to be removed [11].

Even with sufficient arguments for both approaches, in general, the two-stage procedure is seen as the method of choice [12], [24], [26].

Based on our patient data, the aim of this work is to determine the success rate of re-implantation after periprosthetic infections after TKR and to analyze whether there is evidence for an optimal time for re-implantation, the number of revision surgeries or the type of spacer used.

## Patients and methods

We retrospectively evaluated all patients with the ICD-10 diagnosis T84.5 (infection and inflammatory reaction due to artificial joint) who were treated in our clinic with multi-stage revision surgery between 01.09.2005 and 31.12.2013. The collection of patient data was carried out based on electronic health records in SAP IS-H (Siemens AG Healthcare Sector, Erlangen, Germany) as well as archived patient records.

A total of 34 patients could be determined from the patient cohort with infection after TKR. 35 re-implantations of a TKR were performed within the above-mentioned period after remission of infection. This group of patients consisted of 53% (18) male and 47% (16) female patients with a mean age at the time of re-implantation of 72.2 years (54 to 85 years). The left knee was involved in 54.3% (19) and the right knee was involved in 45.7% (16) of the cases, one patient presented with bilateral infection of the TKR. In 83% of knee replacements (29) and 82% of patients (28) a single germ could be detected, whereas a mixed infection was detected in three cases. The most frequently found microbes were *Staph. aureus* (37.5%), and *Staph. epidermidis* (25%). The list of the individual microorganisms and the development of microorganisms at the time of re-infection are shown in Table 1 (Tab. 1).

**Figure 1:**
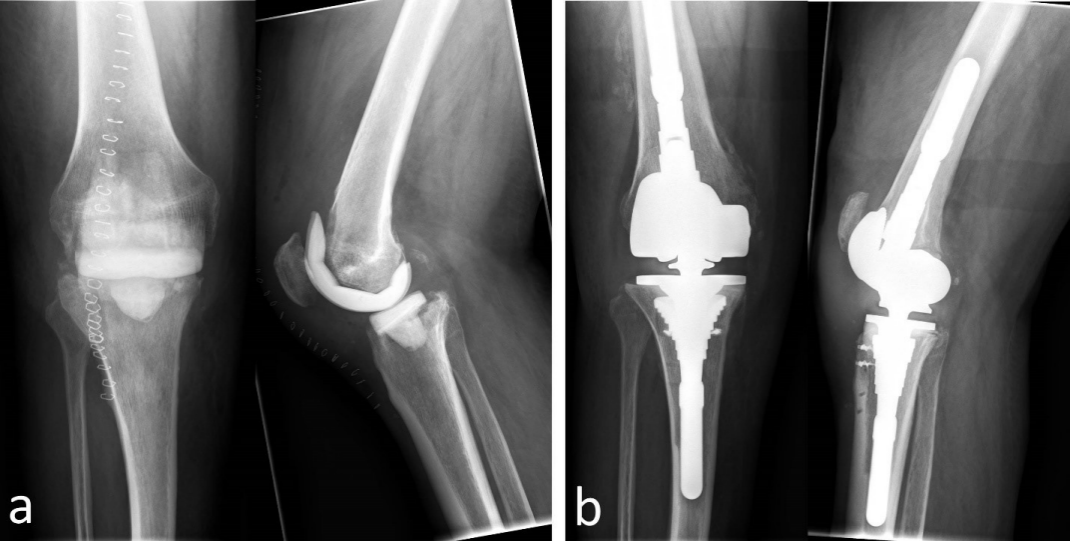
62-year-old patient with TKR infection right. a) Radiograph of the knee anterior-posterior and lateral after implantation of a movable spacer (knee spacer AGC Style Company BiometOrthopedics Inc. Warsaw, U.S.A.). b) X-ray of knee anterior-posterior and lateral after re-implantation of revision-TKR (Typ LCS revision, Company DePuy Synthes, West Chester, PA, U.S.A.)

**Figure 2:**
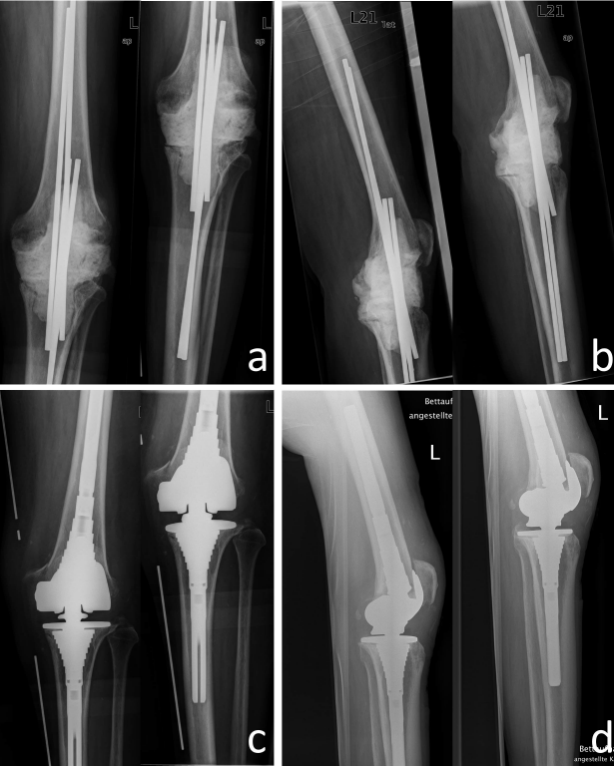
79-year-old patient with TKA infection left. a) Radiograph of the knee anterior-posterior and b) lateral after temporary arthrodesis with intramedullary fixation with titanium rods and cement mantle. c) X-ray knee anterior-posterior and d) lateral after re-implantation of a revision-TKR (Typ S-ROM, Company DePuy Synthes, West Chester, PA, U.S.A.)

Surgical debridement and artificial joint explantation were carried out in all cases with temporary implantation of antibiotic containing cement spacer. In 85.7% (30) of the cases we used mobile spacers; in 14.3% (5) we introduced fixed spacers with intramedullary fixation (Figure 1 (Fig. 1) and Figure 2 (Fig. 2)). In those cases with mobile spacers we used the spacers produced by AGC Style Company Biomet Orthopedics Inc. Warsaw, USA. The system consists of a femoral shape size of 60 to 75 mm in 5 mm increments and a tibial mold size of 65 to 80 mm also available in 5 mm increments. Both are each filled with 80 g of cement and adapted to the local anatomy (Figure 1 (Fig. 1)). As intramedullary/fixed placeholder carbon or metal rods were intramedullary introduced and covered and surrounded with cement according to the defect size in the knee. In those cases we used Copal G+C Heraeus Medical GmbH (Wehrheim, Germany), each with 1 g of gentamicin and clindamycin applied to 40 g cement.

4–6 weeks of systemic antibiotics were administered according to the antibiogram. Subsequently, after cessation of antibiotic treatment, the exclusion of infection was carried out performing a single joint puncture and microbiological and histological examinations. This was followed by the individual re-implantation of total knee.

The statistical evaluation was carried out with the spreadsheet software Microsoft Excel (Microsoft Corporation, Redmond, USA).

## Results

Re-implantation of total knee took place after an average of 5.9 months (on average 178 days, minimum: 20 days; maximum: 21 months). The follow-up period averaged 18 months (12 to 52 months).

Out of the 35 re-implantations a reinfection occurred in 31.4% (11) cases in which surgical revision was performed. Explantation was carried out in 4 patients and a cement spacer was introduced. In 5 cases, an inlay exchange was performed with lavage, 2 were exclusively treated arthroscopically. In the remaining 2 patients an arthrodesis was performed.

Further, in 2 cases (5.7%) we reported non-infectious complications which also had to be treated surgically (tissue necrosis and a mechanical complication), after which the two patients had an uncomplicated postoperative course. 

The time to re-infection in the above mentioned cases was averaged 9.18 months (average of 275 days, min: 9 days; max: 41.1 months). Compared to the primary infection the spectrum of germs has significantly changed, even though *Staph. aureus* (33.3%) and *Staph. epidermidis* (20%) dominated, as shown in Table 1 (Tab. 1). In 3 cases, the same strain responsible for the primary infection was identified, in 8 cases, however, the pathogen changed.

In 6 patients out of those 11 patients in whom re-infected TKR (54.5%) occurred, no further complications were recorded. In 5 patients (45.5%), however, revision surgery had to be performed after an average of 105.8 days (7 to 311 days). In 4 cases, partial or complete explanation of the implant with consequent implantation of a cement spacer was carried out. In one patient, a knee arthroscopy was performed with lavage.

Concerning the cement spacer, we introduced spacers with medullary fixation in 5 cases and mobile spacers in 30 cases. In 3 cases out of the 5 (60%) treated with intramedullary spacers a re-infection occurred in the further course. In the group of patients treated with the movable spacers, we observed reinfection only in 8 of 30 (26.7%) patients.

Looking at the outcome of the re-implantation as a function of time between TKR-removal and replacement we found that those patients in which re-implantation was carried out after an average period of 6.79 months (0.67 to 20.6 months with an uncomplicated course) had no further complications. Among patients with a subsequent infection, the time to re-implantation of the TK was shorter: 4.47 months (0.73 to 8.93 months). A listing of the time period from explantation to re-implantation and the resulting revisions and re-infection is shown in Table 2 (Tab. 2).

As shown in Table 2 (Tab. 2), complications were documented only in re-implantation within the first 9 months after explantation. After a period of more than 9 months no more re-infections were recorded in our patients and hence no further revision operations had to be carried out.

In Table 3 (Tab. 3) the re-infection rate according to the number of revisions performed in the time-interval between explantation and re-implantation is shown. The number of revisions seems to have played no significant role concerning the incidence of reinfection. 

## Discussion

Periprosthetic infection of the knee is a complication that has been confronting us ever since TKR was carried out. The therapeutic options continued to develop, but there are different views in current literature and neither a uniform therapeutic method nor a clearly successful therapeutic scheme have been suggested [22], [30]. In some sources, the one-stage procedure is considered the treatment of choice for periprosthetic infections, especially in cases of known pathogens with resistance patterns, in which topical and systemic antibiotics are allowed, so as to prevent patients from undergoing repeated surgery with its additional risk of complications and even more burdens on the patient [10], [11], [34]. Macario et al. compared the cost of one- and two-stage TK-revisions and showed that a one-stage procedure for the in-patient hospital costs is lower by 24% than that of two-stage revision; rehabilitation was excluded in this context [23]. 

On the other hand, Romanò et al. reported that the average rate of remission of infection was greater after two-stage surgery than after one-stage [30]. Therefore, and in view of further literature, the two-stage procedure for the treatment of periprosthetic infections is considered as the “gold standard” [2], [6], [21], [24], [26]. The two-stage procedure is clearly preferred in situations with clinically manifest infections without proven pathogen or unknown germs, wherein a targeted topical antibiotic (e.g. as in cement) is uncertain. The two-stage procedure is also recommended in case of microorganisms with resistance to antibiotics or in cases which do not allow topical antibiotic therapy, or in chronic infections, mixed infections and multi-resistant germs [10], [34].

In the two-stage TK-revision the optimal time of the re-implantation is of considerable importance. If we re-implant too soon, there is a risk that the periprosthetic infection has not yet been completely remedied. Waiting too long, however, is often accompanied by a number of repeated soft tissue revision surgeries leading to fibrotic changes of the soft tissues with deteriorated final functional results [10], [11]. There is no definitive statement in literature concerning the optimal interval between explantation and re-implantation and the information concerning the duration of the implant-free interval vary considerably [22]. In literature, periods ranging from 2 to 6 months as well as from 2 weeks to several months are reported [22], [26]. This period also depends on certain patient-specific factors, such as general condition of the patient, wound conditions, perhaps the presence of fistula, the extent of infection, the nature of the germ layer and resistance determination as well as the success of antibiotic therapy [18]. According to Friesecke, the duration of periprosthetic knee infection in almost 70% of patients ranges from more than 2 months to over a year [10]. Therefore, it seems that a longer interval between explantation and re-implantation makes sense in order to allow enough time for the infection to heal. Although our study is too small for statistically valid conclusions, we have noticed a lower re-infection rate when re-implantation was carried out after more than 9 months following explantation (Table 2 (Tab. 2)). Concerning Friesecke’s report, it is also apparent that the rate of persistence of infection after single-stage surgery (15%) seems to be too low when compared to other published analysis in which approximately two thirds of patients had to be repeatedly operated on due to persistence of infection [10], [11]. 

In our study, we found no significant differences with regard to the number of revision surgeries carried out prior to re-implantation and the rate of re-infection (Table 3 (Tab. 3)). The number of revision surgery (debridement) should always be determined depending on the individual case and the patient specific distinct findings. In some cases, it might be necessary to carry out a series of planned revision surgeries to maximize the chance of infect-free tissue status.

The typical germs responsible for periprothetic infections of the knee are staphylococci, especially *Staph. aureus*, *Staph. epidermidis* and more coagulase-negative staphylococci [21], [26], [31]. In our study, we could also confirm that staphylococci were the most frequently found germs. Furthermore, mixed infections occurred in our patients leading to a longer and tedious course of therapy comparable to the reports described by Claassen et al. and Spiegl et al. [5], [32]. In recent years, the incidence of multi-resistant germs has significantly increased and thus necessitating appropriate antibiotic therapy [31], [32]. In this context, Frommelt provided a good overview of the antibiotic treatment [12].

In our study, we noticed that the type of infectious organism isolated during re-infection was different to the one isolated at the time of primary infection in the predominant proportion of patients (Table 1 (Tab. 1)). It is therefore to be assumed that the general condition of the patient is as essential as the surgical procedure and antibiotic treatment for successful therapy and remission of infection. According to the study by Claassen et al., age, sex, BMI, rheumatoid arthritis, diabetes mellitus, immunosuppression, and permanent anticoagulation seem to have no influence on the success rate of two-stage revision surgery. On the contrary, patients with excessive intake of nicotine, multiple comorbidities, increasing number of operations, multi-resistance germs and mixed infections had increased complications [5], [26]. Generally, there are inconsistent opinions regarding the question of whether the dead space resulting after explantation of the implants of TK is to be filled up by bone cement, interim endoprostheses or rather kept without introducing any space-holder [12], [26]. Benefits of treatment with a spacer are the already mentioned combination with antibiotics, safeguarding the joint space and knee stability, preventing shortening of collateral ligaments in addition to preserving soft tissue tension and thus resulting in relatively good functionality during the endoprosthetic-free period [26], [30]. Adverse effects of a spacer may be an increased abrasion of the spacer material, the dislocation of the placeholder and pain caused by insufficient fixation. Furthermore, there is no uniform approach the kind of spacer (mobile vs. fixed with intramedullary fixation) used [30]. According to Romanò et al. the average rate of remission of infection with a mobile spacer is higher than with a fixed spacer [30]. This also corresponds to the observations we made concerning our patients in this study. 74.1% (20) of the patients we treated using mobile spacers had a complete remission of infection compared to only 50% (4) of those patients we treated using a fixed spacer. Kuzyk et al. came to the conclusion that mobile spacers are as effective in terms of eradication of infection as fixed spacers, but provide the advantage of better tissue tension and thus facilitate re-implantation [19].

## Conclusions

- Following reimplanatation of TK after periprosthetic infection reinfection occurred in approximately 30% of cases.
- In about 2/3 of the cases the infectious microorganism were different compared to those isolated during the primary infection. This indicates the vital importance of individual patient factors.
- Longer intervals between explantation of TK and re-implantation (>9 months) proved to favour remission of infection.
- The number of revision surgeries that were performed prior to re-implantation seems to have no significance on the outcome.
- Mobile spacers appear to favor the outcome compared to fixed spacers, though the sample size in our study is relatively small to enable a valid statement on this matter.

## Limitations

The main limitations of this study are the relatively short follow-up period of approximately 2 years, the retrospective design and the rather small to medium sized group with 34 patients, or 35 re-implantations. Comorbidities and risk factors were not considered in this analysis.

## Notes

### Competing interests

The authors declare that they have no competing interests.

### Authors’ contributions

MG and DZ contributed equally to this work.

MG contributed substantially to devising the study as well as the analysis and interpretation of the data. JB and VG carried out the majority of data acquisition. TP and DZ were involved in drafting the manuscript. AR, CJ and CEH gave their final approval for the version to be published. MM was involved in creating the figures. The final manuscript has been read and approved by all the authors.

## Figures and Tables

**Table 1:**
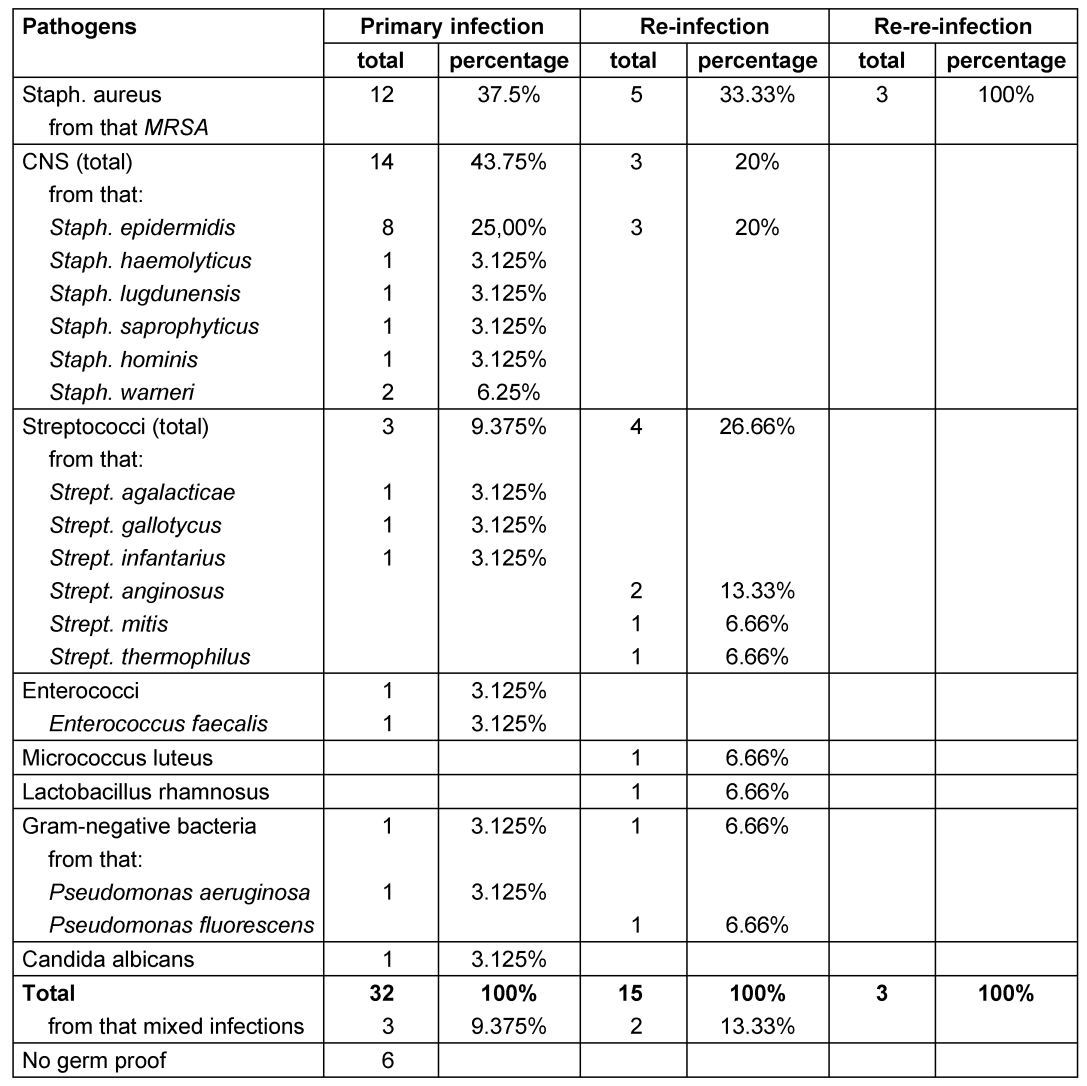
Presentation of pathogens distribution to primary infection, re-infection and re-re-infection (absolute and percentage)

**Table 2:**
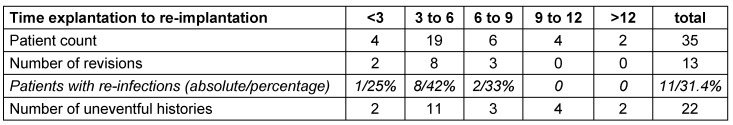
Presentation of period from implanting to re-implantation of TKA and number of revision and re-infections based on the number of patients (absolute and percentage)

**Table 3:**
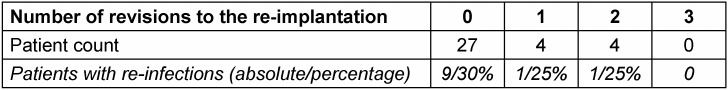
Number of revisions to the re-implantation and number of re-infections based on the number of patients (absolute and percentage)
